# Romosozumab adverse event profile: a pharmacovigilance analysis based on the FDA Adverse Event Reporting System (FAERS) from 2019 to 2023

**DOI:** 10.1007/s40520-024-02921-5

**Published:** 2025-01-14

**Authors:** Luyu Liu, Shaobo Wu, Liangliang Wei, Zhihao Xia, Jiajia Ji, Dageng Huang

**Affiliations:** 1 Department of Spine Surgery, Honghui Hospital, Xi’an Jiaotong University, Xi’an, 710054 Shaanxi China; 2 Department of Orthopaedics, Lanzhou University Second Hospital, Lanzhou, 730030 Gansu China

**Keywords:** Romosozumab, Osteoporosis, Adverse drug reactions, FAERS, Pharmacovigilance

## Abstract

**Objective:**

This study aims to analyze adverse drug events (ADE) related to romosozumab from the second quarter of 2019 to the third quarter of 2023 from FAERS database.

**Methods:**

The ADE data related to romosozumab from 2019 Q2 to 2023 Q3 were collected. After data normalization, four signal strength quantification algorithms were used: ROR (Reporting Odds Ratios), PRR (Proportional Reporting Ratios), BCPNN (Bayesian Confidence Propagation Neural Network), and EBGM (Empirical Bayesian Geometric Mean).

**Results:**

Screening for romosozumab-related AEs (adverse events) included 23 system organ categories (SOCs). PT (preferred terms) levels were screened for adverse drug reaction (ADR) signals. A total of 7055 reports with romosozumab as the primary suspect (PS) and 14,041 PTs induced by romosozumab as PS were identified. Common significant signals of general disorders and administration site conditions, musculoskeletal and connective tissue disorders have emerged. Specifically, unexpected AEs such as gastrointestinal disorder, respiratory, thoracic and mediastinal disorders also occur. Notably, fracture (n = 503, ROR = 107.8, PRR = 103.83, IC = 6.6, EBGM = 97.02) and bone density abnormal (n = 429, ROR = 343.65, PRR = 332.77, IC = 8.08, EBGM = 271.34) exhibited relatively high occurrence rates and signal strengths.

**Conclusion:**

Our study identifies potential new AE signals and provides broader data support for the safety of romosozumab. In clinical application, doctors are provided with a warning to closely monitor adverse reactions to support their rational use in diseases such as osteoporosis.

## Introduction

Osteoporosis is one of the most common bone diseases [1], and the National Institutes of Health (NIH) defined osteoporosis (OP) as a bone disease characterized by a decrease in bone strength and an increased risk of fracture in 2001 [2], which is primarily affected by bone density and bone mass [3]. In people with osteoporosis, bone loss outpaces new bone growth, causing bones to become porous, fragile, and susceptible to breakage, and increasing the risk of fracture [4, 5]. Osteoporosis is an age-related disease that has become one of the leading causes of disability and death in the elderly [6–8]. With an increasingly aging global population, osteoporosis has become a major public health challenge worldwide [9].

Romosozumab is a humanized monoclonal antibody (IgG2) that acts by inhibiting the activity of sclerostin (SOST) to reduce bone resorption and increase bone density while accelerating bone formation [10, 11]. Romosozumab-aqqg (Evenity) is a humanized IgG2 monoclonal antibody targeting sclerostin and is the world's first sclerostin [12]. Sclerostin is an important inhibitor of the Wnt/β-linker protein signaling pathway [13], which is not only closely related to bone metabolism, but also plays a role in a number of other target molecules, including cardiovascular disease and cancer [14]. Sclerostin is closely associated with vascular calcification and is present in several affected diseases such as hyperparathyroidism and atherosclerosis [15].

In April 2019, the FDA approved the osteoporosis treatment for marketing in the U.S. for the treatment of osteoporosis in postmenopausal women at high risk of fracture [16]. In addition, for patients with osteoporosis who have failed or are intolerant to other available osteoporosis therapies, the romosozumab has shown significant efficacy in reducing fracture risk and increasing bone mineral density [17]. Although romosozumab has become one of the most promising therapeutic agents in the clinic due to its significant efficacy advantages in the treatment of osteoporosis, it is important to note that it is also associated with varying degrees of adverse effects, ranging from the occurrence of adverse symptoms in various systems to even death, and the accompanying controversy has continued to draw the attention of researchers to romosozumab adverse events (AEs)[18].

The most common adverse reactions include myocardial infarction, allergic reactions including angioneurotic edema and erythema multiforme, hypocalcemia, osteonecrosis of the jaw, and atypical femur fracture [19–21]. Although available studies have not shown a clear association between romosozumab and cardiovascular events, the European Medicines Agency (EMA) does not recommend the use of romosozumab in patients with myocardial infarction or stroke [22–24]. Also known as a sclerostin inhibitor, romosozumab may affect multiple systems such as heart failure, kidney damage, hypocalcemia, and other related [25, 26]. Headache, joint and muscle pain, and digestive symptoms have also been clinically reported [27, 28]. Also, because the treatment of osteoporosis is a long process, the results of long-term treatment still need to be confirmed [29].

However, there is a lack of large-scale data analysis of systematic and comprehensive real-world and big data-based adverse drug reactions (ADRs) for romosozumab. The presence of other ADRs in the use of romosozumab remains to be investigated. Therefore, this study aims to statistically evaluate the clinical data of romosozumab ADRs from different perspectives to reduce the risk of medication and optimize the therapeutic measures for osteoporosis by using the data mining method of the FDA Adverse Event Reporting System (FAERS) database.

## Methods

### Data sources

The FDA Adverse Event Reporting System (FAERS) database is a publicly accessible database of all adverse event reports that are spontaneously submitted to the US Food and Drug Administration (FDA) for the purpose of collecting uploaded adverse event reports, including medication errors and product quality feedback, from healthcare professionals, pharmaceutical manufacturers, patients and others [30]. The database is updated quarterly and is recognized worldwide as a widely accepted reporting system due to its comprehensive data collection system and strict standardization requirements [31].

The adverse event data collected in this study were obtained from the FAERS database. Given the timing of the drug's launch, reporting files from the second quarter of 2019 through the third quarter of 2023 were downloaded for this study. From these cases, reports in which romosozumab was suspected to be the primary drug associated with ADE were extracted for this study, and the authors obtained a total of 10,611,701 reports for romosozumab. Drug names were standardized through the Medex_UIMA system. The reports we collected included a wide range of information about patients with romosozumab-related adverse events, such as date of report, age and sex of the patient, reporter and region, and clinical characterization of the outcomes. Serious outcomes primarily included life-threatening events or events resulting in hospitalization, disability, or death.

### Signal filtering

The FAERS data file contains seven types of records: patient demographics and management information (DEMO), drug information (DRUG), adverse event coding (REAC), patient outcomes (OUTC), reporting source (RPSR), treatment start and end dates for reported drugs (THER), indications for drug administration (INDI), and deleted cases. All data were imported into MySQL 8.0 for preprocessing, and we obtained 8,016,909 DEMO reports, exiting to remove 1,128,133 duplicates and retaining only the most recent report based on that date, yielding 6,888,776 reports for subsequent analysis. The authors assigned role codes for AEs, including primary suspected use (PS), secondary suspected use (SS), concomitant use (C), and interacting use (I). After integrating 346,999,911 drug information data (DEMO) and 206,545,939 REAC data, to improve accuracy, we selected PS (primary suspect) and extracted 7055 romosozumab-related AER data and 14,041 PTs induced by romosozumab.

**Fig. 1:**
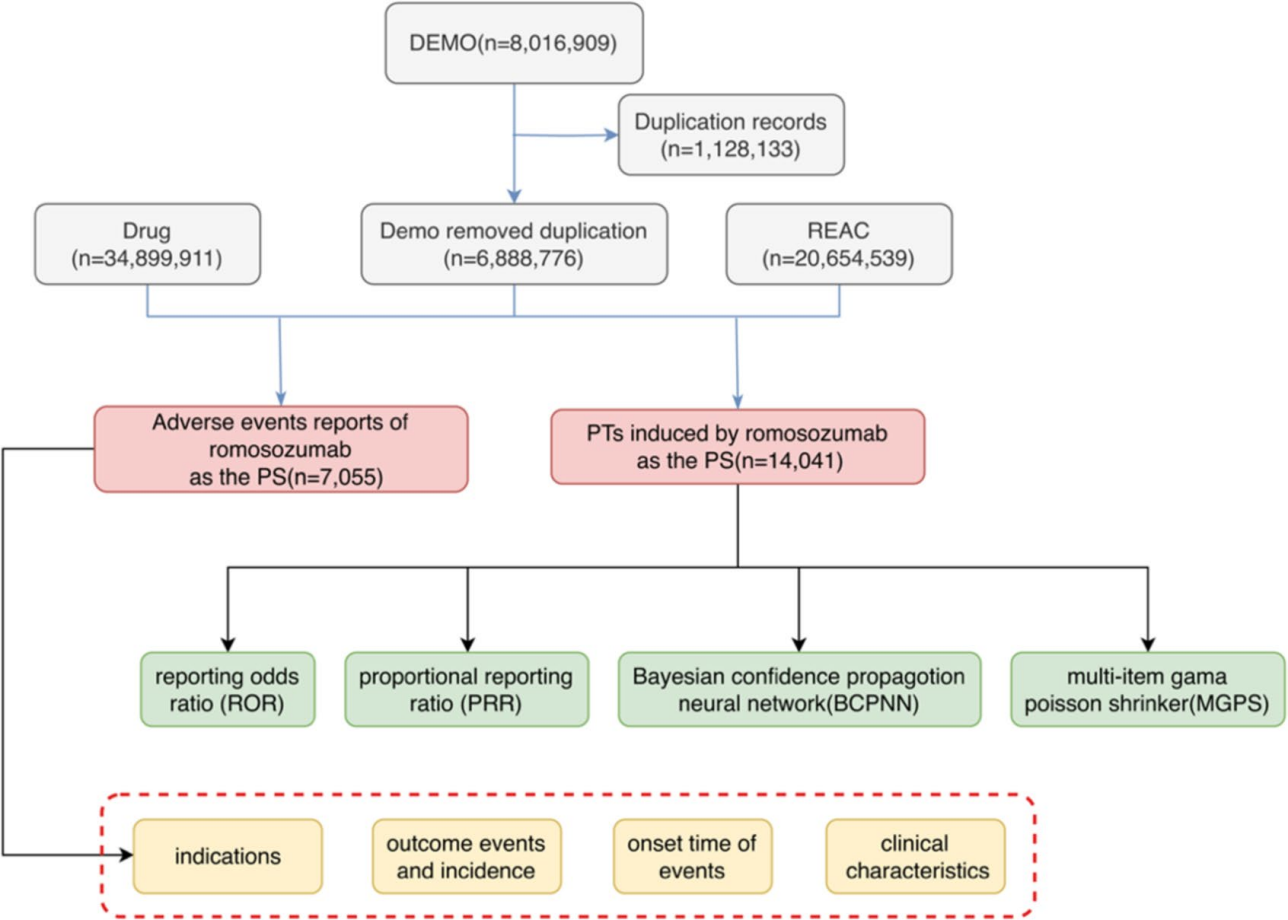
The flow diagram of selecting romosozumab-related AEs from the FAERS database

The structural hierarchy of MedDRA terminology is divided into five levels: System Organ Class (SOC), High-Level Group Term (HLGT), High-Level Term (HLT), Preferred Term (PT) and Lowest Level Term (LLT). The authors used systematic organ classification (SOC) to code, classify, and localize signals and to analyze the specific SOCs involved in the adverse event signals. In an initial screening of the FDA adverse event report database, we selected the top 30 preferred terms (PTs) with the highest number of reports and accurately analyzed the frequency of adverse events and the level of signal strength using four algorithms. The general flow chart of this study is shown in the following figure (Fig. 1).

### Signal analysis algorithms

Proportional Disproportionality Analysis is considered to be a fundamental tool for identifying methods to analyze drug-related adverse events in pharmacovigilance studies, based on the principle of using a four-cell tabulation method to analyze the degree of correlation between a drug and an AE by comparing the ratio of frequencies observed in exposed and unexposed populations. In this study, Proportional Reporting Ratio (PRR), Reporting Odds Ratio (ROR), Bayesian Confidence Propagation Neural Network (BCPN) and Empirical Bayesian Geometric Mean (EBGM) were used to detect drug AE signals.

In this study, a combination of the four algorithms was applied and cross-validated to leverage the strengths of the different algorithms, integrate data from multiple sources and validate the results from multiple perspectives, reduce bias and improve specificity, and detect more potentially rare ADEs.

The proportional reporting ratio (PRR) is a statistical method for signal detection of adverse drug reactions (ADRs) based on unbalanced proportions [32]. In a 2 × 2 table, the proportion of all ADRs for a particular drug for a particular situation is divided by the same proportion for all other drugs in the database [33]. Proportional reporting rate (PRR) is the evaluation of signal generation in the surveillance database. A PRR of 1 indicates that the reported frequency of romosozumab-related adverse effects is as high as that in the control group. A PRR greater than 2 indicates that the reporting rate of romosozumab-related adverse effects was more than twice that of the control group [34].

PRR is an assessment of signal generation in a surveillance database, but does not estimate relative risk [35]. However, when the spontaneous reporting database is considered as the source data for case–control studies, the use of the reporting odds ratio (ROR) allows careful selection of controls to reduce bias [36]. Based on the usual principles of control selection in case–control studies, the ROR determines the inclusion and exclusion conditions for reporting. It has an advantage over the PRR in that it allows estimation of relative risk.

The BCPNN algorithm applies the Bayesian discriminant principle to calculate the information component (IC) based on the classical four-grid table, which reports the disproportionality index between the drug and the expected number of adverse events [37]. Its confidence interval (CI) reflects the strength of the association between the drug and the adverse reactions, which can be considered as an adverse reaction signal if the lower limit of the 95% CI of the IC is greater than 0 [38, 39]. The EBGM method is the most conservative of the four methods in this study and may be more suitable for pharmacoepidemiologic studies [40].

In this study, a valid ADR result was considered to be selected when all four of the above algorithms were satisfied with positive signals. The formulas for the four-grid tabulation method are shown in Table 1, and the specific formulas and threshold for the four algorithms are shown in Table 2. Higher values for the four algorithms indicate a stronger signal strength, which suggests a stronger association between the drug and the occurrence of an adverse event. All romosozumab-related data were processed and statistically analyzed using R 4.3.2, and other software.

**Table 1:** Fourfold table of disproportionality method

	Target drug ADEs	Other drug ADEs	Total
Target drug	a	b	a + b
Other drugs	c	d	c + d
Total	a + c	b + d	N = a + b + c + d

variable "a" represents the number of individuals experiencing expected adverse events after romosozumab exposure, variable "b" represents the number of individuals experiencing non-target adverse events after romosozumab exposure, variable "c" represents the number of individuals facing target adverse events after non-romosozumab exposure, and variable "d" represents the number of individuals experiencing non-target adverse events after non-romosozumab exposure. The total count N is given by the sum of a, b, c, and d (N = a + b + c + d). ADEs:Adverse effects

**Table 2:** ROR, PRR, BCPNN, and EBGM methods, formulas, and thresholds

Method	Formula	Threshold
ROR	${\text{ROR}} = \frac{{{\text{a }}/{\text{ c}}}}{b / d}$	a ≥ 3 and 95%CI (lower limit) > 1
	$$SE\left( {lnROR} \right) = \sqrt {\frac{1}{{\text{a}}} + \frac{1}{{\text{b}}} + \frac{1}{{\text{c}}} + \frac{1}{{\text{d}}}}$$	
	$$95\% CI = {\text{ e}}^{{\ln \left( {ROR} \right) \pm 1.96se}}$$	
PRR	${\text{PRR}} = \frac{{{\text{a }}/{ }\left( {{\text{a}} + {\text{b}}} \right)}}{{c / \left( {c + d} \right)}}$	a ≥ 3 and 95%CI (lower limit) > 1
	$$SE\left( {lnPRR} \right) = \sqrt {\frac{1}{{\text{a}}} - \frac{1}{{{\text{a}} + {\text{b}}}} + \frac{1}{{\text{c}}} - \frac{1}{{{\text{c}} + {\text{d}}}}}$$	
	$$95\% CI = {\text{ e}}^{{\ln \left( {PRR} \right) \pm 1.96se}}$$	
BCPNN	${\text{IC}} = {\text{log}}_{2} \frac{{p\left( {x, y} \right)}}{p\left( x \right)p\left( y \right)} = log_{2} \frac{{a\left( {a + b + c + d} \right)}}{{\left( {a + b} \right)\left( {a + c} \right)}}$	IC025 > 0
	$${\text{E}}\left( {{\text{IC}}} \right) = {\text{log}}_{2} \frac{{\left( {a + \gamma 11} \right)\left( {a + b + c + d + \alpha } \right)\left( {a + b + c + d + \beta } \right)}}{{\left( {a + b + c + d + \gamma } \right)\left( {a + b + \alpha 1} \right)\left( {a + c + \beta 1} \right)}}$$	
	$${\text{V}}\left( {{\text{IC}}} \right) = \frac{1}{{\left( {ln2} \right)^{2} }}\left[ {\frac{{\left( {a + b + c + d} \right) - a + \gamma - \gamma 11}}{{\left( {a + \gamma 11} \right)\left( {1 + a + b + c + d + \gamma } \right)}} + \frac{{\left( {a + b + c + d} \right) - \left( {a + b} \right) + a - \alpha 1}}{{\left( {a + b + \alpha 1} \right)\left( {1 + a + b + c + d + \alpha } \right)}} + \frac{{\left( {a + b + c + d + \alpha } \right) - \left( {a + c} \right) + \beta - \beta 1}}{{\left( {a + b + \beta 1} \right)\left( {1 + a + b + c + d + \beta } \right)}}} \right]$$	
	$${\upgamma } = {\upgamma }11\frac{{\left( {a + b + c + d + \alpha } \right)\left( {a + b + c + d + \beta } \right)}}{{\left( {a + b + \alpha 1} \right)\left( {a + c + \beta 1} \right)}}$$	
	$${\text{IC}} - 2{\text{SD}} = {\text{E}}\left( {{\text{IC}}} \right) - 2 \sqrt {{\text{V}}\left( {{\text{IC}}} \right)}$$	
EBGM	${\text{EBGM}} = \frac{{a\left( {a + b + c + d} \right)}}{{\left( {a + c} \right)\left( {a + b} \right)}}$	EBGM05 > 2
	$$SE\left( {lnEBGM} \right) = \sqrt {\frac{1}{{\text{a}}} + \frac{1}{{\text{b}}} + \frac{1}{{\text{c}}} + \frac{1}{{\text{d}}}}$$	
	$$95\% CI = {\text{ e}}^{{\ln \left( {EBGM} \right) \pm 1.96se}}$$	

## Results

### Basic characteristics of romosozumab-related ADEs

The data for this study includes a total of 8,016,909 adverse event reports from the FAERS database from Q2 2019 through Q3 2023, covering the timeframe of romosozumab’s launch. Of these, romosozumab was identified as the primary suspected adverse drug event (ADE) in 7055 reports. There were significantly more female than male patients in the romosozumab adverse event reports (71.06% vs. 6.05%).

In terms of age, reports of age greater than 75 years were most common. However, a significant amount of data (58.61%) did not provide age information, which prevented us from gaining a deeper and more comprehensive understanding of the relationship between age and adverse events. Notably, the majority of reports (46.01%) were from healthcare professionals. The vast majority of reports came from the United States (52.36%) and Japan (41.55%). In terms of route of administration, subcutaneous injection accounted for 49.88% of the total. In terms of adverse clinical outcomes due to the drug, apart from those not clearly described, adverse events leading to increased hospitalization and prolonged hospital stay were the most common (30.32%), followed by death (8.23%). 10.90% of patients experienced an adverse reaction to tocilizumab within seven days of dosing, most commonly after more than 60 days of dosing (14.35%), and 64.49% of patients experienced an adverse reaction at an unknown time. Details are shown in Table 3.

**Table 3:** Demographic characteristics of reports with romosozumab from the FAERS database (2019 Q2–2023 Q3)

Variable	Total
Year	
2019	642 (9.10)
2020	1110 (15.73)
2021	1382 (19.59)
2022	2305 (32.67)
2023	1616 (22.91)
Sex	
Female	5013 (71.06)
Male	427 (6.05)
Unknown	1615 (22.89)
age_yr	76.00 (69.00, 83.00)
age_yrQ	
< 18	3 (0.04)
18 ~ 45	28 (0.40)
45 ~ 65	386 (5.47)
65 ~ 75	859 (12.18)
> = 75	1644 (23.30)
Unknown	4135 (58.61)
wt	53.00 (44.00, 62.80)
Reporter	
Physician	3246 (46.01)
Pharmacist	1849 (26.21)
Consumer	1826 (25.88)
Other health-professional	90 (1.28)
Unknown	44 (0.62)
Reported countries	
United States	3694 (52.36)
Japan	2931 (41.55)
Other	245 (3.47)
Netherlands	72 (1.02)
Germany	61 (0.86)
United Kingdom	52 (0.74)
Route	
Other	3520 (49.89)
Subcutaneous	3519 (49.88)
Intramuscular	16 (0.23)
Outcomes	
Other serious	3214 (58.00)
Hospitalization	1680 (30.32)
Death	456 (8.23)
Life threatening	106 (1.91)
Disability	77 (1.39)
Required intervention to prevent permanent impairment/damage	7 (0.13)
congenital anomaly	1 (0.02)
tto	32.00 (2.00, 119.00)
ttoQ	
< 7	382 (10.90)
7 ~ 28	174 (4.96)
28 ~ 60	186 (5.31)
> = 60	503 (14.35)
Unknown	2261 (64.49)

### Signals detection associated with romosozumab

In the present study, by analyzing the signal intensity of romosozumab at the system organ classification (SOC) level, we found that the adverse reactions associated with this drug were mainly directed to 23 SOCs, as shown in Table 4. The study revealed that the three most common socs were injury, poisoning and procedural complications (n = 3052, ROR = 2.10, PRR = 1.85, IC = 0.89, EBGM = 1.85), general disorders and administration site conditions (n = 2652, ROR = 1. 09, PRR = 1.07, IC = 0.1, EBGM = 1.07), and musculoskeletal and connective tissue disorders (n = 1474, ROR = 2.18, PRR = 2.05, IC = 1.03, EBGM = 2.05), consistent with romosozumab as a systemic skeletal analog.

**Table 4:** Signal intensity of ADEs of romosozumab reported at the system organ classification (SOC) level in the FAERS database

soc	Case reports	ROR (95% CI)	PRR (95% CI)	IC (IC025)	EBGM (EBGM05)
Cardiac disorders	707	2.63 (2.44, 2.84)	2.54 (2.35, 2.75)	1.35 (1.24)	2.54 (2.39)
Musculoskeletal and connective tissue disorders	1474	2.18 (2.06, 2.3)	2.05 (1.97, 2.13)	1.03 (0.96)	2.05 (1.96)
Injury, poisoning and procedural complications	3052	2.1 (2.02, 2.19)	1.85 (1.78, 1.92)	0.89 (0.83)	1.85 (1.79)
Investigations	1115	1.4 (1.31, 1.49)	1.36 (1.28, 1.44)	0.45 (0.36)	1.36 (1.3)
Nervous system disorders	1120	1.09 (1.03, 1.16)	1.09 (1.03, 1.16)	0.12 (0.03)	1.09 (1.03)
General disorders and administration site conditions	2652	1.09 (1.04, 1.13)	1.07 (1.03, 1.11)	0.1 (0.04)	1.07 (1.03)
Vascular disorders	260	0.99 (0.87, 1.12)	0.99 (0.88, 1.11)	− 0.02 (− 0.19)	0.99 (0.89)
Metabolism and nutrition disorders	257	0.95 (0.84, 1.08)	0.95 (0.84, 1.07)	− 0.07 (− 0.25)	0.95 (0.86)
Ear and labyrinth disorders	52	0.9 (0.69, 1.18)	0.9 (0.68, 1.18)	− 0.15 (− 0.54)	0.9 (0.72)
Endocrine disorders	25	0.69 (0.47, 1.03)	0.69 (0.47, 1.02)	− 0.53 (− 1.08)	0.69 (0.5)
Renal and urinary disorders	187	0.63 (0.55, 0.73)	0.64 (0.56, 0.73)	− 0.65 (− 0.85)	0.64 (0.57)
Gastrointestinal disorders	656	0.58 (0.53, 0.63)	0.6 (0.55, 0.65)	− 0.74 (− 0.85)	0.6 (0.56)
Infections and infestations	454	0.57 (0.52, 0.63)	0.59 (0.53, 0.65)	− 0.77 (− 0.91)	0.59 (0.54)
Skin and subcutaneous tissue disorders	448	0.53 (0.48, 0.58)	0.54 (0.49, 0.6)	− 0.88 (− 1.02)	0.54 (0.5)
Eye disorders	135	0.5 (0.42, 0.6)	0.51 (0.43, 0.61)	− 0.98 (− 1.22)	0.51 (0.44)
Respiratory, thoracic and mediastinal disorders	303	0.47 (0.42, 0.52)	0.48 (0.43, 0.54)	− 1.06 (− 1.23)	0.48 (0.44)
Neoplasms benign, malignant and unspecified (incl cysts and polyps)	225	0.38 (0.34, 0.44)	0.39 (0.34, 0.45)	− 1.34 (− 1.53)	0.4 (0.35)
Hepatobiliary disorders	44	0.38 (0.29, 0.52)	0.39 (0.29, 0.52)	− 1.37 (− 1.79)	0.39 (0.3)
Immune system disorders	53	0.31 (0.24, 0.41)	0.32 (0.24, 0.42)	− 1.66 (− 2.05)	0.32 (0.25)
Blood and lymphatic system disorders	64	0.27 (0.21, 0.34)	0.27 (0.21, 0.34)	− 1.89 (− 2.24)	0.27 (0.22)
Psychiatric disorders	202	0.26 (0.22, 0.29)	0.27 (0.24, 0.31)	− 1.91 (− 2.11)	0.27 (0.24)
Reproductive system and breast disorders	18	0.2 (0.13, 0.32)	0.2 (0.12, 0.32)	− 2.29 (− 2.94)	0.2 (0.14)
Congenital, familial and genetic disorders	7	0.18 (0.09, 0.38)	0.18 (0.09, 0.38)	− 2.45 (− 3.45)	0.18 (0.1)

Romosozumab is usually well tolerated, our study data suggest that musculoskeletal and connective tissue disorders and injuries, toxicity and surgical complications, and general illness and site of use conditions are the top three potential adverse events. Among the most common adverse reactions are fractures, including bone density abnormal, spinal fracture, femur fracture, spinal compression fracture, femoral neck fracture, compression fracture, radius fracture, pathological fracture, lumbar vertebral fracture, thoracic vertebral fracture, and humerus fracture.

Systemic diseases and sites of administration Various reactions include decreased strength, hard nodules at the site of external application, and underlying disease. Other systems include, renal and urinary disorders, gastrointestinal disorders, and vascular and lymphatic vascular disorders, corresponding to adverse reactions with higher signal intensity are vesicoureteral reflux, dental resorption, and aortic entrapment, respectively.

At the PT level, four algorithms were used in this study to analyze adverse drug reactions and assess compliance with various screening criteria to obtain PT. The top 30 SOCs in terms of number of reports according to the four algorithms are shown in Table 5. The results showed that among injuries, poisonings, and procedural complications, fractures (n = 503, ROR = 107.8, PRR = 103.83, IC = 6.6, EBGM = 97.02) and bone density abnormalities (n = 429, ROR = 343.65, PRR = 332.77, IC = 8.08, EBGM = 271.34) had relatively high incidence and signal strength. Among the other SOCs, the occurrence of pathological fracture, aortic dissection, increased parathyroid hormone in the blood, and marasmus after romosozumab treatment were noteworthy, as they occurred quite frequently and with high signal strength. In addition to the adverse events already mentioned in the specification, this study found decreased serum procollagen type I N-terminal prepeptide, increased tartrate-resistant acid phosphatase, and pseudarthrosis, and although these adverse events were rare, they were all high in frequency and signal strength.

**Table 5:** Top 30 rankings of signal intensity of romosozumab adverse events by EBGM at the PTs level in the FAERS database

soc	pt	Case reports	ROR (95% CI)	PRR (95% CI)	IC(IC025)	EBGM(EBGM05)
Injury, poisoning and procedural complications	Fracture	503	107.8 (98.32, 118.2)	103.83 (94.14, 114.52)	6.6 (6.47)	97.02 (89.83)
Injury, poisoning and procedural complications	Radius fracture	46	106.79 (79.13, 144.11)	106.43 (79.32, 142.81)	6.63 (6.21)	99.28 (77.26)
Injury, poisoning and procedural complications	Atypical fracture	10	83.75 (44.27, 158.43)	83.69 (43.83, 159.8)	6.31 (5.43)	79.22 (46.47)
Injury, poisoning and procedural complications	Femoral neck fracture	78	82 (65.24, 103.07)	81.54 (64.45, 103.16)	6.27 (5.94)	77.29 (63.83)
Injury, poisoning and procedural complications	Compression fracture	51	69.29 (52.3, 91.8)	69.03 (52.46, 90.83)	6.04 (5.64)	65.97 (52.13)
Injury, poisoning and procedural complications	Spinal compression fracture	118	61.1 (50.78, 73.51)	60.57 (50.77, 72.25)	5.86 (5.6)	58.21 (49.86)
Injury, poisoning and procedural complications	Thoracic vertebral fracture	27	42.1 (28.71, 61.75)	42.02 (28.39, 62.19)	5.35 (4.81)	40.88 (29.67)
Injury, poisoning and procedural complications	Lumbar vertebral fracture	36	37.2 (26.71, 51.8)	37.1 (26.59, 51.77)	5.18 (4.71)	36.21 (27.45)
Injury, poisoning and procedural complications	Ulna fracture	7	33.74 (15.95, 71.39)	33.72 (16.01, 71.02)	5.04 (4.03)	32.99 (17.62)
Injury, poisoning and procedural complications	Spinal fracture	147	33.6 (28.51, 39.6)	33.24 (28.42, 38.88)	5.02 (4.79)	32.53 (28.35)
Injury, poisoning and procedural complications	Traumatic fracture	6	33.3 (14.82, 74.81)	33.28 (14.9, 74.33)	5.03 (3.94)	32.57 (16.54)
Injury, poisoning and procedural complications	Femur fracture	139	29.79 (25.16, 35.27)	29.49 (24.72, 35.18)	4.85 (4.61)	28.93 (25.12)
Injury, poisoning and procedural complications	Humerus fracture	27	28.42 (19.41, 41.61)	28.37 (19.55, 41.17)	4.8 (4.26)	27.85 (20.24)
Musculoskeletal and connective tissue disorders	Pseudarthrosis	9	258.61 (127.29, 525.41)	258.44 (127.62, 523.36)	7.78 (6.82)	219.83 (121.48)
Musculoskeletal and connective tissue disorders	Jaw clicking	3	49.94 (15.8, 157.85)	49.93 (15.71, 158.7)	5.59 (4.15)	48.31 (18.44)
Musculoskeletal and connective tissue disorders	Pathological fracture	43	45.09 (33.27, 61.1)	44.95 (33.5, 60.31)	5.45 (5.01)	43.64 (33.84)
Musculoskeletal and connective tissue disorders	Exostosis of jaw	4	38.3 (14.19, 103.37)	38.29 (14.09, 104.04)	5.22 (3.94)	37.34 (16.27)
Investigations	Serum procollagen type i n-terminal propeptide decreased	5	2441.75 (583.47, 10,218.46)	2440.85 (583.65, 10,207.74)	9.84 (8.35)	915.94 (276.49)
Investigations	Tartrate-resistant acid phosphatase increased	4	837.11 (245.02, 2859.97)	836.86 (243.44, 2876.87)	9.06 (7.55)	532.91 (190.63)
Investigations	Bone density abnormal	429	343.65 (309.02, 382.17)	332.77 (301.71, 367.03)	8.08 (7.93)	271.34 (248.27)
Investigations	Blood parathyroid hormone increased	25	61.23 (41.03, 91.39)	61.12 (41.3, 90.45)	5.88 (5.31)	58.71 (42)
General disorders and administration site conditions	Application site induration	3	146.48 (44.7, 480.04)	146.45 (44.3, 484.1)	7.06 (5.56)	133.23 (49.35)
General disorders and administration site conditions	Pre-existing disease	3	37.88 (12.04, 119.2)	37.88 (12.15, 118.06)	5.21 (3.77)	36.95 (14.16)
General disorders and administration site conditions	Physical deconditioning	22	28.23 (18.51, 43.06)	28.19 (18.32, 43.39)	4.79 (4.19)	27.67 (19.44)
Vascular disorders	Aortic dissection	27	49.22 (33.53, 72.25)	49.12 (33.19, 72.69)	5.57 (5.03)	47.56 (34.49)
Renal and urinary disorders	Vesicoureteric reflux	3	47.77 (15.12, 150.86)	47.76 (15.03, 151.8)	5.53 (4.09)	46.28 (17.68)
Nervous system disorders	Ruptured cerebral aneurysm	3	30.31 (9.66, 95.08)	30.3 (9.72, 94.44)	4.89 (3.46)	29.71 (11.41)
Neoplasms benign, malignant and unspecified (incl cysts and polyps)	Osteosarcoma	3	29.49 (9.4, 92.5)	29.49 (9.46, 91.91)	4.85 (3.42)	28.92 (11.11)
Metabolism and nutrition disorders	Marasmus	21	65.26 (42.14, 101.07)	65.16 (42.34, 100.29)	5.96 (5.35)	62.43 (43.29)
Gastrointestinal disorders	Tooth resorption	4	83.71 (30.56, 229.32)	83.69 (30.8, 227.4)	6.31 (5)	79.22 (34.09)

## Discussion

To our knowledge, this is the most systematic and comprehensive detailed pharmacovigilance study of romosozumab-related AEs to date based on the FAERS database. Since romosozumab was approved for marketing in 2019, reports of romosozumab-related AEs have continued to increase each year over a 5-year period due to its widespread use in the marketplace and increased awareness among those in the pharmaceutical industry.

Clinical studies have shown that the use of romosozumab significantly reduces the risk of fractures, particularly hip and spine fractures [41]. In patients with osteoporosis who are at high risk for fractures, romosozumab is effective in improving bone mineral density, reducing the risk of osteoporotic fractures, improving patients' quality of life and reducing associated health risks [42]. Therefore, romosozumab plays an important role in fracture prevention and is widely used in the treatment of osteoporosis patients [43]. Romosozumab is administered subcutaneously once a month for one year [42]. Studies have shown that treatment with romosozumab followed by an antiresorptive drug results in greater increases in bone density and greater anti-fracture efficacy than the reverse order [19, 44].

In our study, significantly more female than male patients used romosozumab (71.06% vs. 6.05%), which is consistent with the indication of romosozumab for the treatment of osteoporosis in postmenopausal women at increased risk of fracture, and may also be related to the trend toward more frequent adverse event reporting in women [45]. It is important to note that many of the data lacked age-specific details, a limitation that affected our understanding of adverse events in different age groups [46]. Future studies will need to collect accurate age data to provide insight into differences in drug response between age groups. In addition, the AE signals appear to be mainly driven by the increased risk in people over 65 years of age. The safety and efficacy of romosozumab in young adults and older men have not been well established [47]. The vast majority of reports came from trends in the United States (52.36%) and Japan (41.55%), which require further investigation to eliminate potential regional or cultural biases. It also serves as a warning to other countries of the need to strengthen ADR monitoring and reporting.

Data from our study showed that patients treated with romosozumab had the highest incidence of all types of fractures during treatment. However, previous clinical trials have shown a decrease in the incidence of fractures with the use of romosozumab. Therefore, it is important to understand that fractures that occur during medication use do not necessarily have a significant causal relationship with adverse effects of the drug. Risk factors for osteoporosis in postmenopausal women include the destruction of bone from previous fractures, therefore, treatment focuses on preventing new fractures in response to the most frequently reported adverse reactions to fractures.

Osteoporosis and fracture risk remain even if BMD is normalized. Continuous monitoring and strategic interventions are necessary if fractures are to be avoided. In addition to medication, fracture prevention equipment includes adequate calcium and vitamin D intake, avoidance of smoking and excessive alcohol consumption, weight-bearing and resistance training, and fall prevention [48].

Our results show that cardiac organ disease is also a more important category in the warning of adverse reactions to romosozumab. At the time of PTs analysis, a number of adverse reactions were reported in the vascular and lymphatic categories of aortic coarctation and had a high signal intensity. The United States (US) Food and Drug Administration (FDA) have previously approved romosozumab, but it carries a black box warning due to elevated cardiovascular risk [49]. Romosozumab acts as a monoclonal antibody against sclerostin [50]. Based on preclinical evidence that inhibition of sclerostin levels is involved in accelerating the vascular calcification process in atherosclerosis, which increases the risk of cardiovascular disease and mortality [51]. Thus, inhibition of sclerostin by romosozumab may lead to dysregulation of serum calcium salts, further contributing to vascular atherogenic calcification [52]. This supports the conclusion of previous safety warnings from the U.S. Food and Drug Administration and the European Medicines Agency that the major safety concern with romosozumab is the potential risk of cardiovascular events and that it is recommended that these drugs be avoided in patients at high risk for cardiovascular disease [53]. Our study suggests that clinicians and patients should carefully evaluate the cardiovascular effects of romosozumab and reduce its use in osteoporotic patients with concomitant severe cardiovascular disease, prevent hypocalcemia prior to injectable administration, or change the type of medication to avoid adverse effects [54, 55]. However, it is important to note that only a small number of cardiovascular events resulted in higher risk signal intensities in our study and therefore the true causal relationship and specific mechanism of action between the use of romosozumab and the resulting cardiovascular events could not be determined. Furthermore, further evidence is needed to clarify the risk–benefit ratio in men.

Clinical trial data suggest that adverse events associated with romosozumab include cardiovascular events, osteoarthritis, and cancer [56]. In our study, abnormal bone mineral density was the most commonly reported adverse event among musculoskeletal and connective tissue disorders, followed by three others: increased blood parathyroid hormone, decreased N-terminal prepeptide of serum type I procollagen, and increased ant tartaric acid phosphatase. Antibodies to sclerostin may be used in the intervention of hyperparathyroidism [57]. Clinical trials have shown that in a subgroup treated with romosozumab, markers of bone formation (serum P1NP) showed transient elevations that peaked after 1 month of treatment. PINP may be useful in assessing differences in bone metabolism and in understanding the efficacy of rehabilitation after drug administration [58]. In terms of drug metabolism, since monoclonal antibodies are too large to be filtered and reabsorbed by the kidneys and are ultimately excreted through the metabolic pathway in the urine, if the kidneys are damaged, romosozumab may not be able to undergo the normal metabolic process, further exacerbating the kidney damage [59]. This is a reminder that further studies are needed to determine the safety of romosozumab, particularly in patients with impaired renal function [60, 61].

## Limitations

Although this study provides a reliable scientific basis for the safety evaluation of romosozumab from multiple perspectives, it should be clear that the FAERS database is a self-reporting system, and reporting bias and bias in the description of results are limitations that cannot be ignored, and there may be sampling bias in countries and regions with a high number of reports. It primarily includes data from the United States and some from Japan, which may limit its generalizability on a global scale. This limitation has been noted in several existing pharmacovigilance studies (e.g., Ekins et al. [62]). Secondly, the FARES database does not assess the severity of adverse events and can only assess adverse events qualitatively, and we initially hypothesized in our study that the occurrence of adverse events would be affected by a variety of factors, including individual differences and the timing of dosing as well as differences in drug effects. For example, the issue of missing age data is a known inherent limitation of the FAERS (FDA Adverse Event Reporting System) database. While age may influence the occurrence of adverse drug reactions (ADRs), it is generally not a decisive factor. Most adverse reactions in FAERS data are attributed to the pharmacological properties of the drugs and administration methods, with age having a more indirect effect [63]. Moreover, the "other" category is typically used for records where the route is unclear or does not fit standard options, possibly due to reporter oversight or the complexity of specific administration methods. This issue is also common in other drug safety monitoring databases, where differences in administration route data may reflect the real complexity of drug use and variations in recording practices across healthcare institutions [64]. Thirdly, the signals of all adverse events represent only statistical correlations, and the exact causality needs to be verified by further investigation. Future studies should combine clinical trials and prospective epidemiologic studies to more accurately assess the safety risk of adverse events with romosozumab.

Despite the limitations, we have undoubtedly provided support for more in-depth studies and subsequent close monitoring in the future, and in particular, some unusual potential risks should be of great concern to drug regulatory agencies.

## Conclusion

This study analyzed the FAERS included by the FDA during the use of romosozumab from multiple perspectives and levels, which provided solid data support and adequate warning of adverse reactions for its safety evaluation. The results suggest that patients and health care professionals should pay close attention not only to common orthopedic disorders when using romosozumab to treat osteoporosis, but also to the presence of patients with impaired cardiovascular, endocrine, and metabolic function, and should monitor the function of these systems in a timely manner. Although some adverse events occur relatively rarely, their high signal intensity cannot be ignored, which need necessary monitor to rationally standardize drug use and reduce the occurrence of adverse events. At the same time, we call for higher quality real-world investigations and causal risk assessment in cases.

## Data Availability

This study was conducted using the FAERS database provided by the FDA. The information, results, or interpretation of this study do not represent the opinions of the FDA. The data generated and analyzed during this study are available from the corresponding author on reasonable request.
